# Sexual Dysfunctions in Parkinson’s Disease and Their Influence on Partnership—Data of the PRISM Study

**DOI:** 10.3390/brainsci12020159

**Published:** 2022-01-25

**Authors:** Thomas Kinateder, Daniela Marinho, Doreen Gruber, Laura Hatzler, Georg Ebersbach, Florin Gandor

**Affiliations:** 1 Movement Disorders Hospital, 14547 Beelitz, Germany; kinateder@kliniken-beelitz.de (T.K.); gruber@kliniken-beelitz.de (D.G.); ebersbach@kliniken-beelitz.de (G.E.); 2 Department of Research and Development, BIAL, 4745-457 Trofa, Portugal; Daniela.Marinho@bial.com; 3 Faculty of Medicine, University of Porto, 4200-319 Porto, Portugal; 4 Department of Neurology, Otto-von-Guericke University Magdeburg, 39120 Magdeburg, Germany; 5 Institute of Sexology and Sexual Medicine, Charité—University Medicine Berlin, 10115 Berlin, Germany; Laura.hatzler@charite.de

**Keywords:** Parkinson’s disease, sexual dysfunction, hypersexuality, partnership, quality of life

## Abstract

Background: Sexual dysfunctions (SD) are common but underreported in Parkinson’s disease (PD) and have negative impacts on the quality of life (QoL) and partnership. Methods: We analyzed the data set from the PRISM study for demographics of SD and their influence on quality of life and partnership. Results: 449/861 (52.1%) PD patients reported SD, with male patients being affected more often and having a longer course of disease. The most common SD in men was erectile dysfunction (ED) (*n* = 152), while women’s most frequent complaints were orgasm dysfunction (*n* = 84) and reduced libido (*n* = 81). Hypersexual SDs were reported significantly more often by men. Spousal caregivers of patients reporting inability to relax and enjoy sex and reduced libido indicated a negative influence on the relationship in general. Negative effects on the sexual relationship were reported significantly more often for patients with ED, difficulties with sexual arousal, inability to relax and enjoy sex, and reduced libido. Hypersexual dysfunctions showed no effect on the relationship. Conclusion: SD is a common but underreported problem in the treatment of patients with PD. Due to the negative influence on the relationship and QoL of patients and caregivers, SD should be assessed routinely.

## 1. Introduction

Parkinson’s disease (PD) is a chronic, neurodegenerative disease and clinically characterized by the cardinal motor symptoms of bradykinesia, rigidity, tremor, and postural instability [1]. With a prevalence of 100–300/100,000, PD is the second most common neurodegenerative disorder after Alzheimer’s disease [2,3]. Since age is one of the most important risk factors for developing PD, it is anticipated that by 2040 the number of PD patients will have doubled from currently about 6 million to 12 million, due to the steadily increasing life expectancy [4].

In addition to the characteristic motor features, non-motor symptoms (NMS) such as sleep disorders, affective disorders, gastrointestinal symptoms, or sexual dysfunction have a high prevalence in PD [5,6,7], and their impact on health-related quality of life often exceeds the effect of motor symptoms [8,9].

Sexual dysfunctions (SD) have a high impact on quality of life (QoL) [8,9,10]. In the general population, SD occur with a prevalence of about 43% in women and 31% in men [11]. The prevalence is disproportionately higher in patients with PD. With a prevalence up to 79%, erectile dysfunction (ED) is the most common complaint in PD men [12]. In female PD patients SD is present in up to 87% [13]. Loss of libido is reported to be the most common SD and is 83% twice as prevalent than in the healthy population [12,14,15,16]. SD furthermore can have a negative impact on the partnership [17,18]. Despite the high prevalence and the high impact on QoL and relationship, SD is often underreported and/or underrecognized in everyday clinical practice and not openly addressed, neither by patients nor physicians.

The aim of this study was to analyze the prevalence of SD in an international PD cohort in relation to demographic data and to investigate their impact on the patients’ partnerships.

## 2. Materials and Methods

We analyzed the dataset from The Parkinson’s Real-world Impact assesSMent (PRISM) study [19], which is publicly available after free online registration (https://prism.bial.com (accessed on 18 August 2021)). 

### 2.1. PRISM Study Design

The study design was published previously in detail [19]. In brief, PRISM was an international, observational, cross-sectional survey designed by an international scientific committee in collaboration with The CureParkinson’s Trust (a United Kingdom-based research-driven charity). Patients were recruited in UK, France, Germany, Italy, Portugal, and Spain. Data were collected using an online questionnaire, completed by PD patients and their caregivers. This questionnaire comprised two main sections; the first section was completed from the perspective of the PD patient, and the second was from the perspective of the primary caregiver. Participation in this study was voluntary, including omitting individual questions that a respondent did not wish to answer. Before entering the survey, participants were informed that all information would be treated confidentially and stored securely, as required by General Data Protection Regulation.

### 2.2. Study Population

PD patients and their caregivers were recruited through PD advocacy groups, email, social media campaigns, leaflets, and specialist PD clinics. Since participation was voluntary, it was not possible to actively screen a PD cohort representative of the whole PD population. However, recruitment efforts aimed at reaching the maximum number of PD patients in each country.

### 2.3. Study Assessment

Non-motor symptoms were assessed using the Non-Motor Symptoms Questionnaire (NMSQuest; International Parkinson and Movement Disorder Society, Inc., Milwaukee, WI, USA) [20]. Impulsivity was analyzed with the Questionnaire for Impulsive-Compulsive Disorder in Parkinson’s Disease (QUIP), assessing problems related to gambling, hypersexuality, hyperconsumption (both buying and eating), dysregulated PD medication intake, and hobbyism [21]. Questions relating to sexual relationships were taken from the Medical Outcomes Study Sexual Functioning Scale (MOS-SFS) [22]. Sociodemographic data, comorbidities, pharmacological treatment, the use of healthcare resources, and the impact of PD on employment, family relationships, sexual relationships and impulse control behavior were obtained using structured questionnaires. Sensitive questions (e.g., relating to sexual functioning) were optional and placed in a separate section at the end of the survey, where it was clearly indicated that these could be completed by the patient or caregiver alone.

### 2.4. Evaluation

In this sub-analysis of the PRISM study, we exploratively evaluated demographic data and data concerning SD and their possible influence on the partnership, which were drawn from the following questions of the questionnaire:

- Question 83: Non-motor Symptoms Questionnaire (answering options dichotomously ‘yes’ or ‘no’)
  ○ statement 18: Feeling less interested in sex or more interested in sex
  ○ statement 19: Finding it difficult to have sex when you try
- Question 84: Questionnaire for Impulsive-Compulsive Disorder in Parkinson’s Disease (answering options dichotomously ‘yes’ or ‘no’)
  ○ statement 2: Sex (compulsive urges)
- Question 86: Medical Outcomes Study Sexual Functioning Scale (answering options scaled from ‘not a problem’ to ’very much a problem’)
  ○ statement 1: Lack of sexual interest
  ○ statement 2: Unable to relax and enjoy sex
  ○ statement 3: Difficult in becoming sexually aroused
  ○ statement 4: Men only: Difficulty obtaining or keeping an erection
  ○ statement 5: Women only: Difficulty in having an orgasm

Increased libido was not asked directly in the questionnaire and was therefore extracted from questions 83 (statement 18) and 86 (statement 1); an increased libido was assumed in patients who answered statement 18 of question 83 (‘Feeling less interested in sex or more interested in sex’) with ‘yes’ and at the same time answered statement 1 of question 86 (lack of sexual interest) with ‘no’.

For analysis of SD’s impact on the partnership, we analyzed questions 97 and 100 from the questionnaire section for caregivers/partners:

- Question 97: Has your relationship with the person with Parkinson’s suffered because of their illness? This is in general, taking into consideration all aspects of your relationship (answering options scaled from ‘not at all’ to ’extremely’).
- Question 100: Has your sexual relationship with the person with Parkinson’s suffered because of their illness? (answering options dichotomously ‘yes’ or ‘no’).

### 2.5. Statistical Analysis

Statistical analysis was performed using Microsoft Excel (Microsoft, Seattle, WC, USA) and the open-source software R (version 4.0.5, Novustat, Wollerau, Switzerland), considering *p* < 0.05 as the level of significance. Continuous variables were presented as median (first-third quartile), whereas categorical variables are depicted as frequencies and percentages. Normality of distribution was assessed by using the Shapiro-Wilk test. In the presence of not-normally distributed data, nonparametric statistical tests were applied. The Wilcoxon rank-sum test was used to compare continuous variables, whereas the χ^2^-test with continuity correction was used to assess for statistical differences in proportions. Correlations were calculated with Spearman’s rank correlation coefficient.

## 3. Results

### 3.1. Demographics of Sexual Dysfunctions in General

Demographic data regarding SD from 861 patients (418 female (48.5%); 433 male (50.3%)) who participated in the PRISM survey between April 2019 und July 2019 are shown in Table 1. Four patients (0.5%) indicated their gender as “other”, three patients (0.3%) preferred not to reveal their gender, and another three patients (0.3%) did not give any information. These ten patients were excluded in the following evaluation.

There was no age difference between men (median [IQR]: 58 (23–26,55–65) years) and women (57 (23–24,55–66) years). However, women had a shorter disease duration compared to men (6 (3–10) years vs. 6 (3–12) years; *p* < 0.05). Information on SD was not provided by all participants. An amount of 311 of 433 (71.8%) male patients and 284 of 418 (67.9%) female patients answered at least one question concerning sexuality. In total, 449 patients (52.1% of the total cohort) complained of SD with a significantly higher proportion of male patients (263 (60.7%) men vs. 180 (43.1%) women; *p* < 0.0001).

A total of 201 (23.3%) patients (130 (31.1%) female and 67 (15.5%) male) denied SD based on NMS- and QUIP-answers. Of these, only 58 (6.7%) patients (34 (8.1%) females, 24 (5.5%) males) answered the MOS-SFS-questionnaire. None of these 58 patients indicated the presence of SD in this questionnaire.

### 3.2. Sexual Dysfunctions in Detail

**Figure 1:**
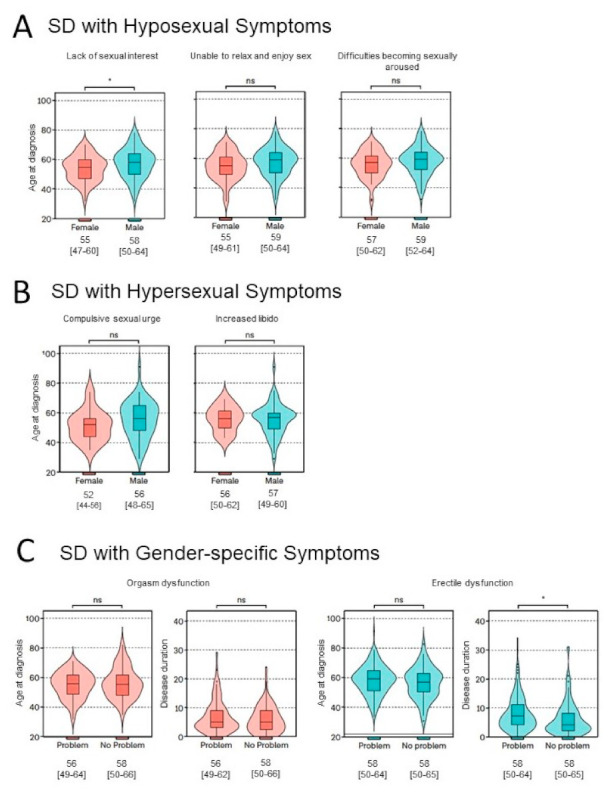
Demographics of sexual dysfunctions in detail. Median age at diagnosis (years) and interquartile range [IQR] are shown below the plots; ns, not significant; * significant correlation at *p* < 0.05; statistical test: Wilcoxon rank-sum test.

The demographic data for each recorded SD are displayed in Figure 1. The absolute numbers of patients answering the respective statement (‘responders’) and more detailed numbers can be found in the Appendix A (Table A1 and Table A2).

With 69.7% (*n* = 152) amongst the responders or 57.6% of all men reporting SD, erectile dysfunction (ED) was the most frequent SD complaint amongst male PD patients. Male patients with ED had a significantly longer median disease duration (7 (4–11) years) than male patients without ED (6 (3–12) years; *p* < 0.05). Amongst women, orgasm dysfunction (OD) was the most frequent SD, reported by 50.9% of the responders or 46.8% of all women reporting SD.

SD concerning hyposexuality in PD patients were disturbed sexual arousal (*n* = 185, 47.7%), the inability to relax and enjoy sex (*n* = 177, 45.4%), and lack of sexual interest (*n* = 174, 44.2%), respectively. The latter was more frequently reported by women than men (female *n* = 81 (45.0%) vs. male *n* = 91 (34.5%); χ^2^ < 0.05), but when reported, men were older than women (male 59 (24–26,55–64) vs. female 57 (23–26,28,55–62); *p* < 0.05).

SD regarding hypersexuality were increased libido and compulsive sexual urges. In total, 17.3% of all patients with SD revealed increased libido, with men significantly more often affected than women (male *n* = 55 vs. female *n* = 19, χ^2^ < 0.0001). Compulsive sexual urges were reported in 15.9% of all patients with SD and significantly more often reported in men (male *n* =55 vs. female *n* = 13; χ^2^ < 0.0001).

### 3.3. Influence of Sexual Dysfunctions on Partnership

SD that had a negative influence on the partnership in general were the inability to relax and enjoy sex (χ^2^ < 0.01) and lack of sexual interest (χ^2^ < 0.01; Table 2). A negative impact on the sexual relationship due to SD was indicated more often from partners of patients with ED (χ^2^ < 0.01), difficulties with sexual arousal (χ^2^ < 0.001), inability to relax and enjoy sex (χ^2^ < 0.001), and lack of sexual interest (χ^2^ < 0.01). The presence of hypersexual disorders showed no influence on the partnership.

## 4. Discussion

To the best of our knowledge, this is the first study using a dataset of an international, observational, cross-sectional study to assess sexual dysfunctions (SD) in patients with Parkinson’s disease (PD) and the influence of SD on the partnership in a large cohort of 861 PD patients.

SD is a frequent but still underrated non-motor symptom of PD [12,14,23,24,25]. Various studies have shown that SD occurs significantly more often in patients with PD than in healthy controls [13,16,26]. In the normal population, the prevalence of SD is around 45% for women and approximately 33% for men, with a high variability across studies [11,27,28]. In our study, 52.1% of the total cohort reported problems with sexuality. Assessing only patients that answered at least one sexuality-related question of the questionnaire, 74.6% (female: 63.4%; male: 84.5%) complained of SD. These numbers are in line with previous studies in significantly smaller cohorts reporting the prevalence of SD in PD patients at about 70% [16,29]. 

In our cohort, significantly more male than female PD patients reported SD. These findings are congruent with the literature. Kovács et al. and Martinez-Martin and colleagues found a higher proportion of men reporting SD, while the rate of NMS concerning mood and sleep was higher in women [30,31]. This difference of NMS prevalence between male and female PD patients is not fully understood. One possible explanation could be differences in the nigrostriatal dopaminergic innervation [32,33] and therefore disparities in the process of dopaminergic denervation. Furthermore, gonadal hormones, especially estrogen, have an influence on the nigrostriatal system [34]. Distinct gonadal hormone levels in men and women could therefore lead to gender differences in the clinical presentation of PD, including non-motor symptoms [35]. In addition, there is evidence of gender-based differences in the gene expression in dopaminergic neurons, e.g., involving upregulation of a-synuclein and PINK-1 genes in male and of maturation and signal transduction genes in female PD patients [36]. Gender may also influence the clinical phenotype of PD, indicating that the nature of PD genetic factors and even the response to therapy may be gender-dependent [37]. Furthermore, studies suggest gender-specific differences in the effect of mitochondrial dysfunction, which seems to have a greater impact in male than female PD patients [38].

With 69.7%, erectile dysfunction (ED) was the most common SD in male PD patients in our cohort, which is in line with previous reports on smaller cohorts, where the prevalence of ED ranges between 68.4–79% [12,39]. This highly exceeds the prevalence in the healthy population, where ED in men is reported in 6.6–22.5% [27,40]. Patients reporting ED had a significantly longer course of disease than patients without. This highlights the fact that age is one of the major risk factors for the development of ED. Numerous studies show that the prevalence of ED increases with older age [39,41]. Shamloul et al. describe an increasing prevalence leading from 2–9% for men between 40 and 49 years to a prevalence of 50% to 100% for men over 70 years of age [42]. 

The most common complaint of female PD patients in our cohort was orgasm dysfunction in 50% and a decreased sexual desire in 48%, respectively, which again highly exceeds the prevalence in the normal population [27,40] and further underlines the importance of actively addressing these symptoms in PD women.

In our study, we also recorded symptoms of hypersexuality, which often occurs as a complication of dopaminergic therapy whereby the use of dopamine agonists in particular increases the risk for hypersexual behavior [43]. A distinction can be made between simple increase in sexual desire and hypersexuality in the context of an impulse control disorder (compulsive sexual urge). While a simple increase in sexual desire can be unproblematic, compulsive sexual urges can lead to conflicts in the partnership or, in severe cases, result in potentially dangerous behavior with criminal consequences due to disturbed impulse control [44]. In our cohort, 17.3% of patients with SD reported increased libido, and 15.9% reported a compulsive sexual urge. Both symptoms were more frequently reported by male PD patients, which is concordant with previous results [43,45,46]. The prevalence of hypersexuality in PD patients is difficult to assess due to the lack of clear diagnostic criteria. Weintraub et al. report a prevalence of up to 10% [47,48]. A systematic review of Codling et al. describes a prevalence between 2–4% [45]. However, the number of unidentified cases is presumably higher, and the prevalence is likely to be underestimated. A study from Switzerland showed a high discordance of the perception of impulse control disorder habits between PD patients and caregivers, especially regarding hypersexuality. While in a cohort of 150 PD patients, 17% of patients reported hypersexuality, and 55% of the caregivers stated hypersexual behavior of their PD partners [49].

The results of our study confirm that SD is a common symptom in PD patients. In addition, non-motor symptoms including sexual dysfunction have a negative impact on quality of life (QoL) [9,10,46]. Yet, there are no studies addressing the influence of SD on the relationship of PD patients and their partners. Importantly, a healthy partnership is a key contributor to QoL [50]. We therefore assessed the influence of SD on the relationship in general and in sexual terms and identified the inability to relax and enjoy sex and lack of sexual interest to have a significant influence on the partnership in general. While other SD certainly also have an impact on the partnership, these two statements have a stronger focus on emotional and interpersonal issues than, e.g., ED or orgasm dysfunction, and might therefore be better suited to capture problems in the relationship in general. As expected, all SD had a negative influence on the sexual partnership, with orgasm dysfunction just missing a level of significance.

In our study, hints of hypersexual symptoms had neither an impact on the partnership in general nor on the sexual partnership, which seems counterintuitive. A possible explanation is the method of data acquisition, since data on hypersexuality were extracted indirectly. Furthermore, our data do not allow for a classification into different degrees of severity. Since a satisfactory sexual life is possible (or even enhanced) in the presence of mildly increased libido or mild compulsive sexual urge, their presence can be experienced as less of a burden on the partnership than hyposexual SD. Nevertheless, there is overall agreement that hypersexuality can influence QoL [10,45].

The negative impact of SD on the partnership and QoL of both patient and partner implies that these symptoms should be routinely recorded by the treating physician. While the prevalence of NMS in PD is estimated to affect every other PD patient [51,52], the prevalence of SD might be even higher because of the intimacy of this topic and the embarrassment to address this issue. Yet, NMS including SD are still underappreciated in the treatment of PD patients [23,24,53], since the clinical focus usually lies in the assessment and therapy of the obvious and objectifiable motor symptoms of PD. The results of this study underline the importance of systematically assessing SD in clinical routine.

We understand that not all participants of the PRISM cohort answered the questions on SD (602 of 861 patients, 69.9%). Furthermore, information from partners/caregivers was only available from 233 of 861 (27.1%). However, we present data from, to our knowledge, the biggest PD cohort that provided information also on SD and their impact on QoL and partnership. It would be desirable to prospectively collect data to investigate the influence of SD on the relationship longitudinally, which should be addressed in future studies. Moreover, we did not assess other factors, e.g., comorbidities including cognitive impairment, nursing care, financial burden, etc., that might also have a negative impact on the partnership. Furthermore, the PRISM study collected data via online surveys, which might lead to a selection bias with recruitment of a younger and cognitively fitter patient cohort. A German survey on the use of technology showed a higher willingness to use digital technology and media in PD patients compared to age matched controls. However, this readiness in PD patients was age dependent [54].

## 5. Conclusions

In summary, we present data from the yet biggest PD cohort that provided information on SD and their impact on QoL and partnership. Our study shows the high prevalence of SD in PD and a negative impact on quality of life. It further shows a negative influence of SD on the partnership. Our study underlines the importance of regularly addressing SD in PD patients, since SD symptoms such as erectile dysfunction can be treated. The occurrence of SD in PD patients is therefore often suitable for medical or psychological interventions, which eventually leads to alleviation of SD symptoms and thereby improving QoL and the partnership. 

## Figures and Tables

**Table 1:** Demographics of sexual dysfunctions in general.

		Total	Female	Male	Significance
All patients					
	N	861	418	433	
	%	100.0%	48.5%	50.3%	
Age at diagnosis (years)	N	827	402	418	
	Median [IQR]	58 (49–65)	57 (49–66)	58 (51–65)	ns
Disease duration (years)	N	813	397	410	
	Median [IQR]	6 (3–11)	6 (3–10)	6 (3–12)	*p* < 0.05 *
Patients answering at least one question concerning sexuality					
	N	602	284	311	
	% total	69.9%	67.9%	71.8%	
Age at diagnosis (years)	N	580	272	303	
	Median [IQR]	58 (50–65)	58 (50–66)	59 (51–65)	ns
Disease duration (years)	N	572	269	298	
	Median [IQR]	6 (3–10)	5 (3-10)	6 (3–12)	*p* < 0.05 *
Patients reporting any kind of sexual dysfunction					
	N	449	180	263	*p* < 0.001 *
	% total	52.1%	43.1%	60.7%	
Age at diagnosis (years)	N	428	169	256	
	Median [IQR]	58 (49–64)	56 (49–63)	59 (50–65)	ns
Disease duration (years)	N	422	167	252	
	Median [IQR]	6 (3–11)	5 (3–10)	7 (4–12)	ns
Patients denying any kind of sexual dysfunction in NMSQuest and QUIP					
	N	201	130	67	ns
	% total	23.3%	31.1%	15.5%	
Age at diagnosis (years)	N	197	128	66	
	Median [IQR]	60 (52–67)	60 (54–67)	59 (49–65)	ns
Disease duration (years)	N	195	127	65	
	Median [IQR]	5 (3–9)	5 (3–8)	5 (2–10)	ns
Patients denying any kind of sexual dysfunction in NMSQuest, QUIP and MOS-SFS					
	N	58	34	24	ns
	% total	6.7%	8.1%	5.5%	
Age at diagnosis (years)	N	58	34	24	
	Median [IQR]	57 (51–67)	58 (54–68)	56 (47–63)	ns
Disease duration (years)	N	58	34	24	
	Median [IQR]	4 (2–8)	4 (2–7)	4 (2–8)	ns

IQR, interquartile range; N, number of patients; NMSQuest, Non-Motor Symptoms Questionnaire; QUIP, Questionnaire for Impulsive-Compulsive Disorder in Parkinson’s Disease; MOS-SFS, Medical Outcomes Study Sexual Functioning Scale; ns, not significant; * Significant correlation at *p* < 0.05; statistical test: Wilcoxon rank-sum test.

**Table 2:** Influence of sexual dysfunctions on the relationship.

Erectile Dysfunction							
	Does the Relationship Suffer?					Does the Sexual Relationship Suffer?	
Severity	Not at all	Slightly	Moderately	Very Much	Extremely	No	Yes
Very much a problem (*n*)	6	7	13	5	6	7	28
Somewhat of a problem (*n*)	6	3	5	1	0	4	9
Little of a problem (*n*)	7	3	2	0	0	7	5
No problem (*n*)	5	3	5	2	0	8	4
	Χ^2^ = 0.1916					Χ^2^ < 0.01 *	
Orgasm dysfunction							
Very much a problem (*n*)	1	0	3	0	0	2	2
Somewhat of a problem (*n*)	3	0	5	2	0	3	7
Little of a problem (*n*)	4	6	3	0	0	8	5
No problem (*n*)	11	4	4	2	0	14	3
	Χ^2^ = 0.0579					Χ^2^ = 0.0569	
Difficulties in becoming sexually aroused							
Very much a problem (*n*)	4	5	8	3	3	3	19
Somewhat of a problem (*n*)	3	6	5	2	0	3	11
Little of a problem (*n*)	14	8	7	1	1	17	13
No problem (*n*)	22	9	18	5	2	31	18
	Χ^2^ = 0.2752					Χ^2^ < 0.001 *	
Unable to relax and enjoy sex							
Very much a problem (*n*)	2	3	8	1	2	1	14
Somewhat of a problem (*n*)	4	7	8	5	4	10	16
Little of a problem (*n*)	8	7	5	0	0	8	10
No problem (*n*)	29	9	18	4	0	35	18
	Χ^2^ < 0.01 *					Χ^2^ < 0.001 *	
Lack of sexual interest							
Very much a problem (*n*)	1	2	6	5	3	1	15
Somewhat of a problem (*n*)	3	4	9	3	2	6	12
Little of a problem (*n*)	9	10	9	0	1	14	14
No problem (*n*)	29	11	17	6	1	34	22
	Χ^2^ < 0.01 *					Χ^2^ < 0.01 *	
Increased libido							
Yes (*n*)	13	7	11	3	1	13	17
No (*n*)	29	20	30	11	6	42	46
	Χ^2^ = 0.8869					Χ^2^ = 0.8378	
Compulsive sexual urges							
Yes (*n*)	5	7	13	4	3	9	19
No (*n*)	63	46	47	33	8	57	79
	Χ^2^ = 0.1169					Χ^2^ = 0.4543	

* statistical significance.

## Data Availability

The PRISM study and database was funded by BIAL-Portela and Cª, S.A., designed in collaboration with The Cure Parkinson’s Trust, an advocacy group based in the United Kingdom (UK), and reviewed by the PRISM steering committee. The data of the PRISM study is freely available after registration under https://prism.bial.com/ (accessed on 18 August 2021).

## Appendix A

**Table A1:** Responding rates of the evaluated questions.

Responders	Total (*n*)	Female (*n*)	Male (*n*)
Question 83			
Statement 18: Feeling less interested in sex or more interested in sex	602	283	311
Statement 19: Finding it difficult to have sex when you try	600	281	311
Question 84 Compulsive sexual urges	601	283	310
Question 86			
Statement 1: Lack of sexual interest	394	169	220
Statement 2: Unable to relax and enjoy sex	389	166	218
Statement 3: Difficult in becoming sexually aroused	388	165	218
Statement 4: Men only: Difficulty obtaining or keeping an erection	389	169	218
Statement 5: Difficulty in having an orgasm	390	165	220

**Table A2:** Demographics of sexual dysfunctions in detail.

		Total	Female	Male	Significance
Erectile Dysfunction					
	N	152		152	
	% responders	69.7%		69.7%	
	% men with SD	57.6%		57.6%	
Age at diagnosis (years)	N	148		148	
	Median [IQR]	58 (23–26,28,55–64)		58 (23–26,28,55–64)	
Disease duration (years)	N	147		147	
	Median [IQR]	7 (4–11)		7 (4–11)	
Orgasm dysfunction					
	N	84	84		
	% responders	50.9%	50.9%		
	% women with SD	46.8%	46.8%		
Age at diagnosis (years)	N	77	77		
	Median [IQR]	56 (23–28,55–62)	56 (23–28,55–62)		
Disease duration (years)	N	77	77		
	Median [IQR]	5 (3–9)	5 (3–9)		
Difficulties in becoming sexually aroused					
	N	185	71	111	ns
	% responders	47.7%	43.0%	50.9%	
	% total with SD	43.2%	39.4%	42.0%	
Age at diagnosis (years)	N	174	63	109	
	Median [IQR]	58 (23–26,28,55–63)	57 (23–26,28,55–62)	59 (24–26,55–64)	ns
Disease duration (years)	N	174	63	109	
	Median [IQR]	5 (3–9)	5 (3–8)	6 (4–11)	ns
Unable to relax and enjoy sex					
	N	177	68	106	ns
	% responders	45.5%	41.0%	48.6%	
	% total with SD	41.4%	37.8%	40.2%	
Age at diagnosis (years)	N	164	60	102	
	Median [IQR]	57 (23–26,28,55–63)	55 (23–28,55–61)	59 (23–26,28,55–64)	ns
Disease duration (years)	N	164	60	102	
	Median [IQR]	5 (3–10)	5 (3–9)	6 (4–11)	ns
Lack of sexual interest					
	N	174	81	91	Χ^2^ < 0.05 *
	% responders	44.2%	48.0%	41.4%	
	% total with SD	40.65%	45.0%	34.5%	
Age at diagnosis (years)	N	163	73	89	
	Median [IQR]	57 (23–28,55–62)	55 (23–30,55–60)	58 (23–26,28,55–64)	*p* < 0.05 *
Disease duration (years)	N	163	73	89	
	Median [IQR]	5 (3–10)	5 (3–9)	6 (3–12)	ns
Increased libido					
	N	74	19	55	Χ^2^ < 0.0001 *
	% responders	18.9%	11.2%	25.0%	
	% total with SD	17.3%	4.4%	12.9%	
Age at diagnosis (years)	N	73	19	54	
	Median [IQR]	57 (23–28,55–61)	56 (23–26,28,55–62)	57 (23–28,55–60)	ns
Disease duration (years)	N	71	19	52	
	Median [IQR]	7 (4–10)	4 (3–10)	7 (4–11)	ns
Compulsive sexual urges					
	N	68	13	55	Χ^2^ < 0.0001 *
	% responders	11.3%	6.7%	17.7%	
	% total with SD	15.9%	3.0%	12.9%	
Age at diagnosis (years)	N	65	12	53	
	Median [IQR]	55 (23–30,54–64)	52 (23–30,52–56)	56 (23–28,30,55–65)	ns
Disease duration (years)	N	63	12	51	
	Median [IQR]	9 (4–13)	7 (3–9)	9 (5–13)	ns

IQR, interquartile range, N, number of patients; ns, not significant; * Significant correlation at *p* < 0.05; statistical test: chi square test.
